# Self-reported wellbeing and body image after abdominoperineal excision for rectal cancer

**DOI:** 10.1007/s00384-016-2628-0

**Published:** 2016-08-10

**Authors:** Elisabeth González, Kajsa Holm, Berith Wennström, Eva Haglind, Eva Angenete

**Affiliations:** 1 Department of Surgery, Institute of Clinical Sciences, Sahlgrenska Academy at University of Gothenburg, SSORG - Scandinavian Surgical Outcomes Research Group, Sahlgrenska University Hospital/Östra, SE-416 85 Gothenburg, Sweden; 2 Department of Surgery, Skaraborgs Hospital, Skövde, Sweden

**Keywords:** Rectal neoplasm, Ostomy, Body image, Quality of life

## Abstract

**Purpose:**

Patients with low rectal cancer are often operated with an abdominoperineal excision (APE) rendering them a permanent stoma. The surgical procedure itself, the cancer diagnosis, and the permanent stoma might all affect quality of life. The aim of this study was to explore wellbeing and body image 3 years after APE in a population-based cohort of patients.

**Methods:**

All patients with rectal cancer operated with an APE between 2007 and 2009 were identified using the Swedish ColoRectal Cancer Registry. A total of 545 patients answered a questionnaire 3 years after surgery. Two open-ended questions were analyzed with a mixed method design using both qualitative and quantitative content analysis. Main themes and sub-themes on wellbeing and body image were identified.

**Results:**

Three main themes were identified: bodily limitations, mental suffering, and acceptance. Bodily limitations included other symptoms than stoma-related problems. A majority of patients expressed acceptance to their situation regardless of bodily limitations and mental suffering. However, 18 % did not describe any acceptance of their current situation.

**Conclusions:**

Most patients expressed acceptance reflecting wellbeing 3 years after APE for rectal cancer. There is, however, a subset of patients (18 %) who describe bodily limitations and mental suffering without acceptance and who require further support. Many aspects of the portrayed bodily limitations and mental suffering could be prevented or treated.

**Trial registration:**

NCT01296984.

## Introduction

Rectal cancer is common and surgery with curative intent is the main treatment, sometimes with the addition of (chemo) radiotherapy [1]. In some cases, the rectal tumor is situated so low in the rectum that it is impossible to perform an anastomosis. The patient will then undergo an abdominoperineal excision (APE) which will render them a permanent colostomy. The surgical procedure itself is associated with morbidity that both in the short and long term may affect quality of life (QoL) [2, 3]. It is not fully known if the colostomy has a negative effect on quality of life and results have been divergent [4–9]. We recently reported that most patients did not feel limited by their stoma 3 years after surgery [10]. This is supported by Feddern et al., but patients with symptoms from their stoma may have problems with acceptance [11].

Studies have indicated that a person’s body image may be of importance regarding depression and anxiety shortly after treatment for colorectal cancer [12]. A small study from Brazil indicated that the stoma itself was associated with a poor body image [13]. On the other hand, a larger study from North America and Europe indicated that many patients still have a positive body image despite their stoma [14]. There are also indications that quality of life improves over time [15], but studies on long term bodily image and acceptance are scarce.

The aim of this study was to describe the body image and wellbeing in patients at least 3 years after their abdominoperineal resection for rectal cancer using a qualitative analysis of answers to open-ended questions in a quality of life questionnaire.

## Materials and methods

### Study design

All patients with rectal cancer operated with abdominoperineal excision during 2007–2009 were identified through the Swedish ColoRectal Cancer Registry (SCRCR) [16] which has a coverage of 97 % and a good internal validity [17]. The cohort has been described elsewhere by Prytz et al. [3, 18] and Asplund et al. [2]. Patients who are alive, able, and willing to answer a questionnaire at least 3 years postoperatively were included in the study. A subset of these patients has been included in a study regarding stoma construction by Marinez et al. [10].

Clinical and demographic data on sex, age, body mass index (BMI), the American Society of Anesthesiologists (ASA) physical status classification, neoadjuvant treatment, and tumor stage were retrieved from the registry.

### The questionnaire

The development and validation of this questionnaire has been described in detail elsewhere [2, 19–21]. The process started by in-depth interviews with patients that had undergone treatment for rectal cancer followed by a qualitative analysis. Questions constructed from the qualitative analysis were content validated in a group of professionals from several disciplines, and the questionnaire was then face-to-face validated. The questions were revised and the process continued until there were no uncertainties [2, 20]. The questionnaire comprised 253 questions covering socio-economy, quality of life, urinary as well as sexual and intestinal function 3 years later. It also included open-ended questions and two of those were analyzed in this study; one focusing on wellbeing: “Describe, in your own words, your wellbeing today” and the other focusing on body image: “Describe, in your own words, how you perceive your body today.”

### Data analysis

A mixed method design with both qualitative and quantitative content analysis was applied to the answers of the two open-ended questions in the questionnaire. Qualitative content analysis is a stepwise process of categorization based on the expression of thoughts, feelings, and actions described throughout the text. The analysis was guided by the descriptions of Sandelowski [22] and was performed in three steps.

Qualitative data was condensed and shortened into codes by multiple interpretations and readings of the texts. These codes were grouped into main themes (A, B, and C) and sub-themes. Patients could belong to more than one main theme based on their open-ended answer to the two questions.

### Statistical analysis

Results were presented descriptively. Categorical data was analyzed with Chi-squared test and continuous data with Mann-Whitney or *t* test. Due to the explorative and hypothesis-generating objective of the study, no correction for multiplicity was performed. Statistical analyses were performed using SPSS 21.0 (IBM SPSS Inc. Armonk, NY, USA) and SAS v9 (SAS Institute, Cary, NC, USA)*.*

### Ethical considerations

This study was approved by the regional ethical committee of Gothenburg, EPN 406–10.

The trial was registered at Clinical Trials.gov, identifier: NCT01296984. Acronym:APER.

## Results

**Fig. 1:**
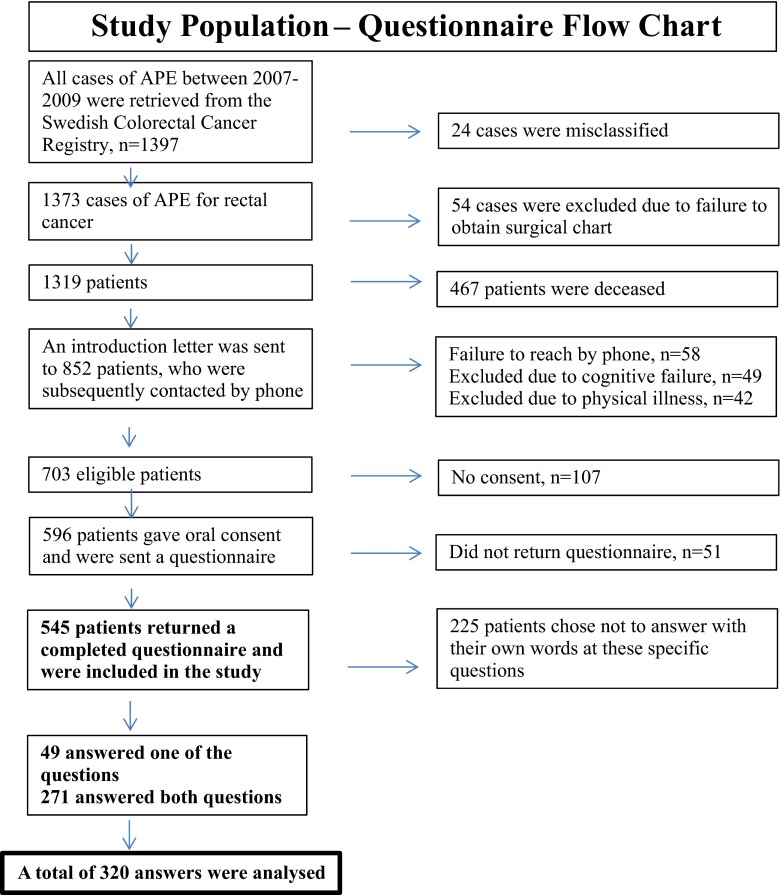
Study population questionnaire flow chart

The total cohort from the SCRCR included 1397 patients. The questionnaire was answered by 545 patients (Fig. 1). The two selected open-ended questions were answered by 320 (59 %) patients responding to one or both questions. Median age was 65 years, 183 men/137 women. There were no differences regarding age, sex, or tumor stage between the group answering both the questionnaire and the open-ended questions and the patients answering only the questionnaire (Table 1). The analysis of the data from the two open-ended questions regarding wellbeing and body image resulted in three main themes: bodily limitations, mental suffering, and acceptanc*e*, together with nine sub-themes*.* These themes reflected the patients’ experience of wellbeing and how they perceived their body 3 years after APE due to rectal cancer (Table 2).

**Table 1:** Demography

Patients	Answered the open-ended questions (*n* = 320)	Did not answer the open-ended questions (*n* = 225)	Total (*n* = 545)
Sex			
Female	137 (43 %)	81 (36 %)	218 (40 %)
Male	183 (57 %)	144 (64 %)	327 (60 %)
Marital status *			
Married or living together in a relationship	231 (74 %)	168 (77 %)	399 (73 %)
Living alone, no relationship	81 (26 %)	49 (23 %)	130 (24 %)
Age median (range Q1:Q3)	65 (59:71)	68 (61:73)	66 (60:73)
BMI median (range Q1:Q3)	25 (22.8:28.1)	25.7 (23.5:28.3)	25.3 (23:28.1)
ASA classification **			
ASA I	77 (25 %)	67 (30 %)	144 (27 %)
ASA II	191 (62 %)	123 (55 %)	314 (59 %)
ASA III	42 (13 %)	31 (14 %)	73 (14 %)
ASA IV	0 (0 %)	2 (1 %)	2 (0 %)
Depression (% No)***	279 (89 %)	183 (85 %)	462 (88 %)
Tumor stage ****			
Stage 0	17 (5 %)	5 (2 %)	22 (4 %)
Stage I	33 (11 %)	15 (7 %)	48 (9 %)
Stage II	101 (32 %)	85 (38 %)	186 (35 %)
Stage III	141 (45 %)	111 (50 %)	252 (47 %)
Stage IV	20 (6 %)	7 (3 %)	27 (5 %)
Pre-operative chemo radiotherapy			
5Gy ×5	201 (63 %)	154 (68 %)	355 (65 %)
1.8Gy × 25	81 (25 %)	41 (18 %)	122 (22 %)
Other	38 (12 %)	30 (13 %)	68 (13 %)
Local recurrence (%)	3(1 %)	3(1 %)	6(1 %)
Type of perineal dissection			
APE	42 (13 %)	29 (13 %)	71 (13 %)
ELAPE	128 (40 %)	94 (42 %)	222 (41 %)
Not stated	150 (47 %)	102 (45 %)	252 (46 %)

*missing (*n* = 16), **missing (*n* = 12), ***missing (*n* = 17), **** missing (*n* = 10)

**Table 2:** Main themes and sub-themes

Main themes	Sub-themes
Theme A “bodily limitations”	1. Stoma-related problems 2. Sexual dysfunction 3. Tiredness/fatigue 4. Other diseases
Theme B “mental suffering”	1. Ashamed of the body 2. Distress
Theme C “acceptance”	1. Unchanged everyday life 2. Positive attitude 3. Gratitude for life

The qualitative analysis revealed three themes and in total nine sub-themes as described below

### Groups of main themes and sub-themes

**Fig. 2:**
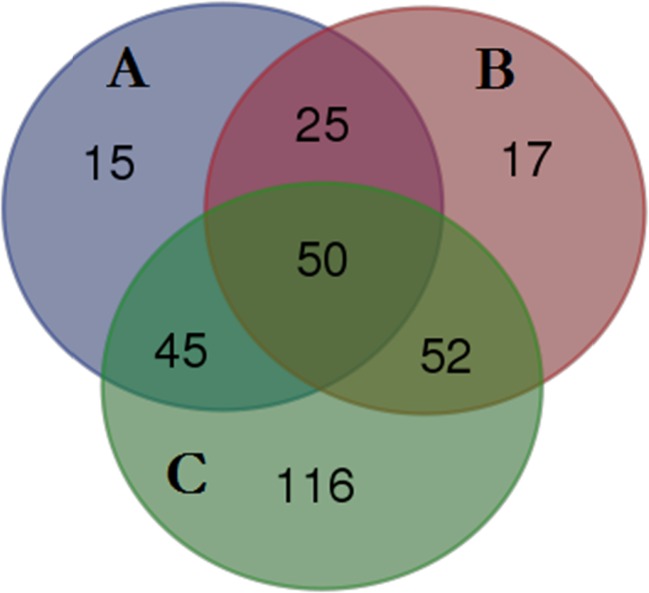
Venn diagram shows the distribution of themes schematically

Groups of main themes (A, B, and C) and combinations of sub-themes resulted in seven groups: A, B, C, AB, ABC, AC, and BC (Table 3, Fig. 2). There were differences between the groups regarding tumor stage, nodal stage (N0 more common in A), and depression (less patients claiming “not depressed” in B (65 %) and AB (48 %)). In total, 147 (46 %) patients had an acceptance of their situation although they expressed bodily limitations and/or mental suffering (ABC, AC, and BC). One hundred and sixteen patients (36 %) experienced acceptance without describing any bodily limitations or mental suffering (C). Only 57 patients (18 %) described bodily limitations and/or mental suffering without acceptance (A, B, and AB).

**Table 3:** Number of patients in each theme and theme combination

Theme and theme combinations*	Number of patients (*n* = 320)
A-bodily limitations	15 (5 %)
B - mental suffering	17 (5 %)
C - acceptance	116 (36 %)
AB - bodily limitations and mental suffering	25 (8 %)
ABC - acceptance although bodily limitations and mental suffering	50 (16 %)
AC - acceptance although bodily limitations	45 (14 %)
BC - acceptance although mental suffering	52 (16 %)

*Each patient belongs to only one theme or combination of themes

### Detailed description of the main themes

#### Theme A: bodily limitations

Bodily limitations involved both small and large changes to the patient’s situation of life and described the various physical and psychological symptoms. This theme was divided into the sub-themes: stoma-related problems, sexual dysfunction, fatigue, and other illnesses/diseases.

##### Sub-theme stoma-related problems

Many patients described stoma-related problems such as parastomal hernia, pain, unpleasant odor, and leakage which made them feel uncomfortable. Additionally, bodily limitations were mainly associated with clothing and social and leisure activities, especially swimming.

I’m worried about stoma leakage. Irritated and sad that many activities I used to do before I cannot do today. I can’t dress the way I want to.

I also have humiliating leakage, am nervous about going to concerts, in case there is noise. I also avoid being seen at the public baths or at beaches.

##### Sub-theme sexual dysfunction

Sexual dysfunction was related to experiences of limitations due to the stoma, as well as physical impairment caused by the cancer treatment. “I often have output leakage, therefore I cannot have an erection or release...” “I’m fine, but due to my large parastomal hernia... I cannot have the same sex life as previously.”.

Two other patients expressed:

I do not have a sexual drive anymore, which I miss sometimes, despite my age. It vanished after surgery.

I suffer from erectile dysfunction after the radiotherapy, and it torments me.

##### Sub-theme tiredness/fatigue

Some patients described that they struggled against fatigue and tiredness. A part of the patients related their tiredness to being older rather than an effect of their treatment or previous cancer.

Unfortunately I would rather stay at home, can’t cope with being as social as earlier …don’t have the strength to do what I want to Affected by ageing. ..have less strength and stamina.

##### Sub-theme other diseases

Patients described how other diseases affected their daily life. They could distinguish the cancer disease from their physical limitations. One of the patients described it like this:

It is bad due to many drawbacks. Have had a hip replacement, had a heart attack 3 months after the stoma surgery, a dislocated shoulder, then a knee fracture, have seriously reduced sight in my left eye, have hearing problems, reduced hearing and very impaired balance …

#### Theme B: mental suffering

Mental suffering included several components that contributed to a feeling of ill-being or grief and was divided into two sub-themes: ashamed of the body and distress.

##### Sub-theme ashamed of the body

Patients described that they were ashamed of their body and felt uncomfortable. Several even described themselves as ugly and mutilated. Many stated that their lives were restricted.

My body is not fit to be seen but functional. UGLY. .. I avoid being seen at the public baths and on beaches…

A female patient described it in this way:

The stoma has also removed my femininity …do not want to be naked in front of my husband. I see a mutilated body in my mirror… an enemy of my intellect.

##### Sub-theme distress

Patients described a kind of discomfort, mostly related to the stoma and a general feeling of malaise. Some patients stated that their symptoms made them feel depressed.

I no longer go to the movies or the theatre in case the stoma falls apart. .. I get paralyzed and sad. .. the most important thing for me is keeping the cancer away. I have no sense of wellbeing today, have to be grateful that I can work. The only time I feel good is when I can go to bed at night.

#### Theme C: acceptance

The need to learn to live with a stoma was common to all the patients; for some, this was easy, for others more difficult. Patients living in a relationship more often expressed an acceptance compared to patients living alone. The main theme “Acceptance”, however, showed that after 3 years, there was an acceptance of the situation. The theme acceptance was divided into three sub-themes, unchanged everyday life, positive attitude, and gratitude for life.

##### Sub-theme unchanged everyday life

This was exemplified as a feeling of health and wellbeing despite the stoma. Some related their acceptance to their advanced age or family.

I have accepted my body, my husband has been so helpful all the time, our children and grandchildren think the stoma bag is exciting. Everything is peaceful.

I want to live as usual and I accept my body despite having lost the rectum…

days can pass without my thinking of having a bag on my stomach…

I try to live like I did before the operation. Don’t feel sorry for myself, but instead so grateful that everything has gone so well so far, and still no recurrence of metastases.

##### Sub-theme positive attitude

We found comments revealing a positive attitude, sometimes through a conscious decision.

...decided from the start to love my stoma. Am grateful that I have it. I’m able to live a normal life.

##### Sub-theme gratitude for life

Many patients described gratitude for being alive. They were grateful for their experiences and felt support and love despite surgery and a stoma.

...am grateful for life and happy for nice little moments. Experience positive things more clearly.

I look at my body with gratitude that it functions so well despite all it has been through.

## Discussion

In our national cohort study, more than 80 % of the patients expressed in their own words that they experienced wellbeing and acceptance of their situation even with symptoms. But it is worrying to find that 18 % of the patients did not accept their present situation 3 years after curative treatment for rectal cancer.

This is one of the few studies that analyze long-term wellbeing and body image in depth after abdominoperineal excision for rectal cancer. The results differ from Feddern et al. as we found that many patients with bodily limitations, in part due to their stoma, still expressed an acceptance [11]. Perhaps this is due to the somewhat different study design with both longer and shorter follow-up in Feddern’s study, but it may also reflect the differences in the questionnaires, as ours included open-ended questions.

In the main theme “bodily limitations”, stoma-related problems emerged as a sub-theme. To some degree this contradicts our previous findings [10], and once again this may be due to the difference in multiple choice questions and open-ended questions. Some patients may find it easier to express their difficulties in their own words, when they do not find an appropriate alternative answer. This illustrates the need for both quantitative and qualitative research in this patient group [23]. Several causes, such as leakage and parastomal hernia, may be preventable or at least possible to treat.

In correspondence with Nichols [14], we also found that bodily limitations were not only due to the stoma; for many patients, other issues seemed to be more important, such as tiredness and fatigue as well as co-morbidity. If this is addressed, perhaps quality of life could be improved.

The strength of this study is the large initial cohort, representing all patients operated in Sweden 2007–2009 which the short collection time should reflect less variation in treatment for all patients. Unfortunately, some patients, who were alive at 3 years, could not be included due to concomitant diseases such as dementia. However, the response rate to our questionnaires, among those able and willing to answer was almost 90 %. It must, however, be remembered that not all patients answered the open-ended questions, rendering the answer frequency for this sub-study 58 %. It still seems reasonable to assume that our cohort represents Swedish patients alive 3 years after abdominoperineal excision for rectal cancer.

The interactive research process is seen as a strength. In the analysis of qualitative data, the authors’ knowledge and experience (preconceptions) accumulated over many years could be regarded as a problem. According to Malterud, however, preconceptions are not the same as bias, unless the researcher fails to acknowledge them [23]. During the process, we repeatedly returned to the purpose of the study and the entire material was analyzed individually and collectively with all authors.

It could be considered a weakness that not all patients who returned the questionnaire answered the open-ended questions. It is possible that the patients who wrote answers to open-ended questions had an advantage compared to those who did not as they were able to express their feelings in writing. A recent randomized controlled study could not, however, find that expressive writing improved the reported quality of life in patients treated for colorectal cancer [24]. A possible limitation is that this study cannot draw any conclusions regarding the body image and wellbeing in patients that were not alive at 3 years.

Patients without acceptance were more often single, which is consistent with previous findings indicating that the social environment assists in finding wellbeing after cancer treatment [25]. It is important to identify this group relatively early in order to intervene and possibly improve their long-term wellbeing. The higher number of patients that were “not depressed” in these groups indicate that perhaps identifying depression early and introducing treatment could improve acceptance. Also, perhaps it would be possible to assist patients further with the help of psychological support and physiotherapists to support patients that do not have sufficient support in their social environment.

In conclusion, most patients expressed an acceptance to their situation indicating wellbeing 3 years after abdominoperineal excision. When reflecting on body image, the stoma is not the only concern; there are also other issues for this group of patients. A subset of patients who do not indicate wellbeing may benefit from further support. The exact design of support measures for this group requires further interventional studies.
